# Auditory Brainstem Evoked Response: response patterns of full-term and premature infants

**DOI:** 10.1590/S1808-86942010000600011

**Published:** 2015-10-19

**Authors:** Raquel Leme Casali, Maria Francisca Colella dos Santos

**Affiliations:** 1 Master's degree, speech therapist, master's degree on child and adolescent health. Center for Pediatric Studies (Centro de Investigação em Pediatria or CIPED), Medical School (FCM) of the Campinas State University (Universidade Estadual de Campinas or UNICAMP); 2 Doctoral degree, speech therapist. Adjunct professor and coordinator of the Speech Therapy Course, Campinas State University (UNICAMP). Campinas State University (Universidade Estadual de Campinas or UNICAMP)

**Keywords:** hearing, electrophysiology, premature, infant

## Abstract

Auditory Brainstem Response (ABR) is important for the early diagnosis of hearing impairment in infants.

**Aim:**

To compare ABR responses in full-term and premature infants; gender and ear were taken into account.

**Methods:**

A cross-sectional prospective cohort study was carried out. We evaluated 36 full-term and 30 premature infants that had passed the Transient Otoacoustic Emissions test, had type A tympanometric curves, and had no risk factor for hearing loss besides prematurity. The evaluations were done from the time of hospital discharge to the third month of life, and consisted of a clinical history, acoustic immittance testing and ABR evaluation.

**Results:**

The comparison of absolute and interpeak wave I, III and V latencies in right and left ears revealed a statistically significant difference at the interpeak I-III. There was no significant gender differences in the comparison of results. Significant difference in wave I, III and V absolute latencies at 80 dB and in wave V at 60 db and 20 db were observed in a comparison of absolute and interpeak latencies between full-term and premature infants. An inverse correlation was found between age and absolute latencies.

**Conclusions:**

The maturity of the auditory system influences ABR responses in infants. To avoid misinterpretation of results, gestational age must be taken into account in the analysis of ABR in pediatric population.

## INTRODUCTION

The mortality rate of high-risk newborn infants has gradually decreased as medical science has advanced, especially in the field of neonatology. These advances have helped increase survival rates especially in premature low birth weight infants. However, the newborn that survive perinatal events are prone to manifest developmental issues such as neurological and/or sensory deficits. This possibility increases as birth weights and gestational ages decrease, which characterizes this population as an at risk group for neurological or sensory disorders, including peripheral and/or central hearing disorders.^1^, ^2^, ^3^

Hearing is paramount for child development; it provides adequate individual integration into a society where oral communication predominates. Hearing disorders may result in language impairment and slower cognitive, intellectual, cultural and social development. Thus, hearing loss should be detected as early as possible so that language and social functioning may develop as normally as possible.

Early detection of hearing loss makes it possible to refer positive cases for medical therapy and rehabilitation programs.^4^, ^5^ The Joint Committee of Infant Hearing (JCIH)^6^ recommends identifying children with hearing loss by universal hearing screening at the moment children are discharged from hospital or within the first month of life. If screening tests are positive, children should be referred to the appropriate medical expert and a speech therapist. A test battery is then undertaken to confirm the diagnosis of hearing loss; this diagnosis should be made by the third month of life, and therapy should be started by the sixth month of life.

The development of universal hearing screening due to JCIH recommendations have increased the workload in speech therapy clinics for evaluating very young children, which do not respond reliably to subjective hearing tests;^2^, ^7^, ^8^, ^9^ thus, objective tools have become extremely useful in this context.

The brainstem auditory evoked potential (BAEP) consist of registering the electrical activity in the auditory system from the inner ear to the brainstem by presenting an acoustic stimulus. This is an easy and non-invasive test for assessing auditory function, and has been widely used in the detection of hearing loss in children, since no patient collaboration is required.^2^, ^7^, ^8^, ^9^ BAEP may also be used for diagnosing auditory threshold changes, characterizing types of hearing loss, identifying retrocochlear or nervous system disorders, and assessing the maturity of the central auditory system in neonates.^10^, ^11^

Auditory system neurological maturity is a two-phase process. The first phase is intrauterine, and is over by the sixth month of gestation; at this point the peripheral auditory pathways are mature. The second phase starts after birth and ends at about 18 months of life; at this point the auditory pathways along the central nervous system up to the brainstem reach maturity.^7^, ^12^, ^13^, ^14^

Several authors have reported that BAEP responses in neonates and nursing infants are affected by the maturity of the auditory system.^7^, ^12^, ^15^, ^16^, ^17^, ^18^, ^19^ The effect of maturity is even more evident in premature infants; thus, the response pattern in these children differs from those in term neonates.^7^, ^15^, ^16^, ^19^, ^20^, ^21^

Studies on the effects of neural maturity of the hearing system in premature neonates are sparse in the Brazilian literature. Gathering normative data is essential because of the importance of diagnostic tests for hearing loss in children and the increased demand for an early identification of hearing loss in neonates and nursing infants. Such data may help learn about the response patterns of a population, differentiating true changes from response patterns, and help analyze the results to raise diagnostic accuracy.

Several studies have underlined the importance of normative data at each healthcare unit, as wave latency values depend on factors such as the stimulus parameter, the device, and population features such as age.^9^, ^17^, ^18^

The purpose of this study was to analyze the response pattern of premature and term neonates and nursing infants to the BAEP, considering the factors gender and ear, and to check the effect of auditory pathway maturity on the electrophysiological responses in a Brazilian population.

## METHODS

A prospective cross-sectional cohort study evaluated 66 male and female children, of which 36 were term neonates (TN) and 30 were premature neonates (PN) according to the World Health Organization classification.^22^

Study subjects were in a rooming-in setting; except for prematurity, they had no other risk factor for hearing loss.^6^

Only lactating infants submitted to neonatal hearing screening (transient otoacoustic emissions test or TOAE, and a type A tympanometric curve, in Jerger and Carvallo's classification system^23^, ^24^) to exclude peripheral (outer and middle ear) auditory disorders were included in this study. An Interacoustics MT10 device with a 226 Hz probe was used for evaluating the middle ear. An Interacoustics OtoRead device was used to assess otoacoustic emissions; this device emphasizes the frequencies 1,000, 1,500, 2,000, 3,000, and 4,000 Hz; a pass/fail criterion was the presence of three frequency bands with a signal-to-noise ratio over 5dB.

Parents or caretakers of children undergoing neonatal hearing screening that met the inclusion criteria were informed about the objectives and importance of the study. Consenting parents or caretakers signed the free informed consent form for inclusion into the study.

The evaluations were carried out between hospital discharge and the third month of life, and included the following procedures: a clinical history, middle ear testing (acoustic immittance), and an electrophysiological evaluation (BAEP).

An Interacoustics Eclipse EP 25 device was used for BAEP testing. This was carried out in a silent, electrically isolated low-lit room. Monaural in-ear phones were used. An intensity of 80 dBHL was used to assess the integrity of auditory pathways and to compare absolute wave I, III and V latency and interpeaks I-III, III-V and I-V among groups. The stimulus was thereafter presented at decreasing intensities (60, 40 and 20 dBHL). White noise (40 dBHL less than the stimulus) was used to mask the contralateral ear.

The test was done with the infant sleeping naturally, usually after a meal. The child was comfortably resting on his or her mother's lap. The skin was cleaned with alcohol and an abrasive paste before applying the conducting gel. Surface electrodes were the active electrode (Fz) and ground (Fpz) on the forehead, and the reference electrodes on the right (M2) and left (M1) mastoids. Impedance between electrodes was less than 3 KOhms, as recommended by the manufacturer.

The BAEP parameters were: rarefaction clicks, 3000 Hz low-pass filter, 50 Hz high-pass filter, 2000 stimuli in total, presentation rate of 19 stimuli/second, 15 ms analysis window. Each recording was made in duplicate to ensure reproducibility.

Presence and absolute latency of waves I, III and V at 80 dBHL and interpeak intervals I-III, III-V and I-V were investigated. Analysis also included the presence and absolute latency of wave V at 60, 40 and 20 dBHL and the inter-ear difference of the wave V absolute latency at all intensities.

The institutional review board approved the study (protocol number 649/2007).

The Kolmogorov-Smirnov statistical test was used to investigate the normal distribution of data. Parametric tests were used if a normal distribution was found; otherwise, non-parametric tests were applied. The chi-square test was used to test the gender homogeneity of groups. The paired t test and paired Wilcoxon test were applied to analyze absolute and interpeak latencies, since these are observations on the same individuals. The sampling unit was the ear for absolute and interpeak latencies without significant differences; otherwise, right and left ears were analyzed separately. The results of absolute and interpeak latencies were studied for males and females using Student's t test or the Mann-Whitney non-parametric test. Males and females were used for comparing results between term and premature infants if absolute and interpeak latencies were significantly different. Student's t test or the Mann-Whitney test were applied to compare wave I, III and V absolute and interpeak latencies and the wave V inter-ear difference in term and premature infants.

Spearman's correlation coefficient was applied to investigate the correlation between gestational age and absolute and interpeak latencies for each group and the complete sample.

The significance level was 5%; data in which statistically significant differences were found are highlighted in bold. The SAS software version 9.1.3 was used for this analysis.

## RESULTS

There were 66 infants, of which 36 were term and 30 were premature. Table 1 shows the sample according to sex, gestational age, and age at the time of testing (gestational age plus post-natal age). The chi-square test showed that the groups were homogeneous with regards to sex (p=0.5561).

**Table 1:** Data of term and premature infants: sex, gestational age, age at test, and gender homogeneity of groups.

		Term	Preterm
Female a	N	19	18
	%	52,8	60
Male a	N	17	12
	%	47,2	40
Age at test	Mean	42,5	40,3
(in weeks)	SD b	1,4	2,6
	Median	42,4	39,5
Gestational age	Mean	39,2	35,7
(in weeks)	SD b	1,1	1
	Median	39,3	36

a Chi-square test p = 0.5561

b Standard deviation

Table 2 shows the comparison of wave I, III and V absolute and interpeak latencies at 80 dBHL and wave V latency at 60, 40 and 20 dBHL between right and left ears of infants. A statistically significant difference was found only at the interpeak I-III.

**Table 2:** Lactating infants according to values (mean, standard deviation and median in milliseconds - ms) and a comparison of absolute wave I, III and V and interpeaks I-III, III-V and I-V latencies at 80 dBHL, and the absolute wave V latency at 60, 40 and 20 dBHL for the right and left ears.

			Right Ear				Left Ear		
Measurement	N	Mean (ms)	Standard Deviation (ms)	Median (ms)	n	Mean (ms)	Standard Deviation (ms)	Median (ms)	p-value
Wave I - 80dBHL	66	1,66	0,32	1,57	66	1,61	0,32	1,50	0,2691^a^
Wave III - 80dBHL	66	4,04	0,31	4,07	66	4,10	0,29	4,05	0,1695
Wave V - 80dBHL	66	6,29	0,43	6,33	66	6,31	0,39	6,39	0,0963^a^
Wave V - 60dBHL	66	6,95	0,37	6,99	66	6,95	0,43	6,95	0,5196^a^
Wave V - 40dBHL	66	7,61	0,37	7,63	66	7,60	0,40	7,63	0,6810^a^
Wave V - 20dBHL	66	8,29	0,29	8,25	66	8,27	0,32	8,25	0,5218
Interpeak I-III	66	2,39	0,35	2,40	66	2,49	0,40	2,55	0,0321^a^
Interpeak III-V	66	2,25	0,38	2,29	66	2,21	0,32	2,23	0,4378
Interpeak I-V	66	4,63	0,41	4,63	66	4,70	0,45	4,72	0,0752^a^

paired t test /

a Wilcoxon's paired test (p<0.05)

No statistically significant difference was found by applying Student's t test and the Mann-Whitney test for comparing wave I, III and V absolute and interpeak latencies at 80 dBHL and wave V latency at 60, 40 and 20 dBHL in males and females; this is shown on Table 3 (p<0.05).

**Table 3:** Values (mean and standard deviation in milliseconds - ms) and comparison of absolute wave I, III and V and interpeak I-III, III-V and I-V latencies at 80 dBHL and the absolute wave V latency at 60, 40 and 20 dBHL for male and female term and preterm infants

					Term						Preterm			
		Male			Female				Male			Female		
Measurement	n	Mean	Standard deviation	n	Mean	Standard deviation	p-value	n	Mean	Standard deviation	n	Mean	Standard deviation	p-value
Wave I - 80dB	34	1,61	0,34	38	1,54	0,23	0,7696^a^	24	1,73	0,37	36	1,69	0,33	0,7235^a^
Wave III - 80dB	34	4,05	0,30	38	3,98	0,29	0,2904	24	4,2	0,29	36	4,10	0,29	0,2199
Wave V - 80dB	34	6,27	0,42	38	6,14	0,42	0,1817^a^	24	6,53	0,28	36	6,35	0,41	0,0786^a^
Wave V - 60dB	34	6,98	0,34	38	6,82	0,39	0,0653	24	7,09	0,33	36	6,99	0,48	0,3542
Wave V - 40dB	34	7,67	0,31	38	7,51	0,43	0,1544^a^	24	7,68	0,32	36	7,60	0,42	0,6252
Wave V - 20dB	34	8,29	0,24	38	8,17	0,35	0,0888	24	8,38	0,32	36	8,32	0,28	0,1013
Interpeak I-III right ear	17	2,4	0,37	19	2,45	0,31	0,6472	12	2,25	0,4	18	2,39	0,35	0,3155
left ear	17	2,49	0,49	19	2,43	0,35	0,6815	12	2,69	0,35	18	2,43	0,38	0,0744
Interpeak III-V	34	2,22	0,42	38	2,16	0,28	0,1496^a^	24	2,33	0,36	36	2,24	0,35	0,3605^a^
Interpeak I-V	34	4,65	0,42	38	4,6	0,40	0,6652^a^	24	4,80	0,46	36	4,66	0,46	0,2123

Student's t test /

a Mann-Whitney test (p<0.05)

Table 4 shows the wave I, III and V absolute and interpeak latencies at 80 dBHL and wave V latency at 60, 40 and 20 dBHL in term and premature infants, and the comparison between groups.

**Table 4:** Values (minimum, mean, maximum and standard deviation in milliseconds - ms) and comparison of absolute wave I, III and V and interpeak I-III, III-V and I-V latencies at 80 dBHL and the absolute wave V latency at 60, 40 and 20 dBHL for male and female term and preterm infants

				Term						Preterm			
Measurement	n	Min. (ms)	Mean (ms)	Max. (ms)	Standard deviation (ms)	Median (ms)	n	Min. (ms)	Mean (ms)	Max. (ms)	Standard deviation (ms)	Median (ms)	p-value
Wave I - 80dBHL	72	1,23	1,57	2,37	0,29	1,50	60	1,30	1,70	2,53	0,35	1,60	0,0193^a^
Wave III - 80dBHL	72	3,37	4,01	4,60	0,29	4,02	60	3,57	4,14	5,00	0,29	4,13	0,0139
Wave V - 80dBHL	72	5,27	6,20	7,27	0,42	6,27	60	5,57	6,42	7,10	0,37	6,47	0,0030
Wave V - 60dBHL	72	6,07	6,89	7,73	0,37	6,90	60	5,97	7,03	7,83	0,42	7,10	0,0489
Wave V - 40dBHL	72	6,43	7,58	8,33	0,39	7,63	60	6,67	7,63	8,33	0,39	7,62	0,5011^a^
Wave V - 20dBHL	72	7,30	8,23	8,87	0,30	8,23	60	7,73	8,34	8,87	0,30	8,33	0,0330
Interpeak I-III right ear	36	1,67	2,42	3,07	0,34	2,40	30	1,67	2,34	3,13	0,37	2,32	0,3198
left ear	36	1,57	2,46	3,17	0,42	2,52	30	1,67	2,53	3,40	0,39	2,59	0,4773
Interpeak III-V	72	1,27	2,19	2,87	0,35	2,23	60	1,40	2,28	3,03	0,35	2,27	0,1667^a^
Interpeak I-V	72	3,80	4,63	5,33	0,41	4,63	60	3,47	4,71	5,50	0,46	4,72	0,1834^a^

Student's t test /

a Mann-Whitney test (p<0.05)

Statistically significant differences were found in a comparison of absolute latencies for waves I, III and V at 80 dB between term and premature infants; higher values were found in premature infants at all intensities compared to term infants. More prolonged wave V latencies were found at 40 dBHL in premature infants, although these differences were not statistically significant. Interpeak I-III, III-V and I-V intervals were higher in premature infants compared to term infants, but were not statistically significant.

Inter-ear wave V differences below or equal to 0.4 ms were observed in 86% of term and 80% of premature infants at 80 dBHL, 83% and 77% at 60 dBHL, 75% and 80% at 40 dBHL, and 89% and 83% at 20 dBHL.

**Figure 1:**
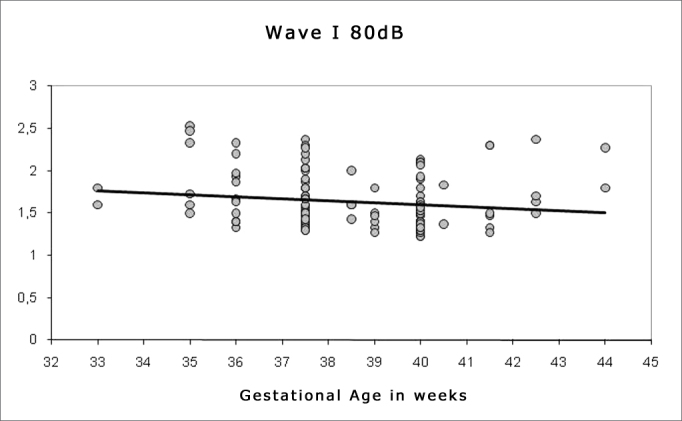
Correlation chart of the gestational age and absolute wave I latency at 80 dBHL. Spearman's correlation coefficient (r= -0.188; p<0.05).

**Figure 2:**
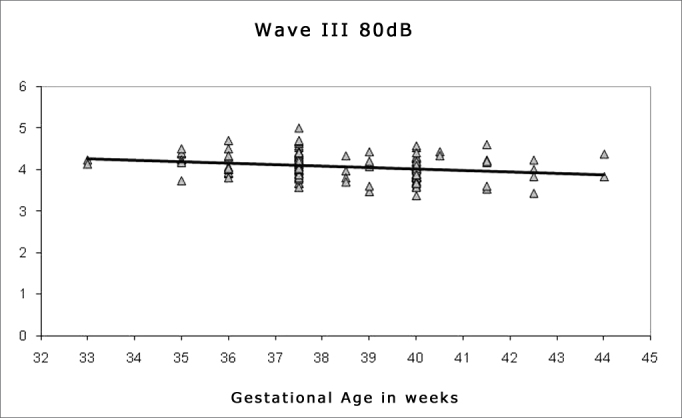
Correlation chart of the gestational age and absolute wave III latency at 80 dBHL. Spearman's correlation coefficient (r= - 0.232; p<0.05).

**Figure 3:**
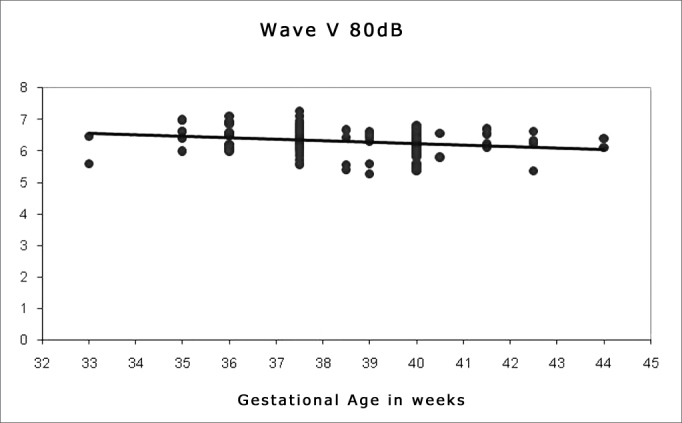
Correlation chart of the gestational age and absolute wave V latency at 80 dBHL. Spearman's correlation coefficient (r=- 0.266; p<0.05).

**Figure 4:**
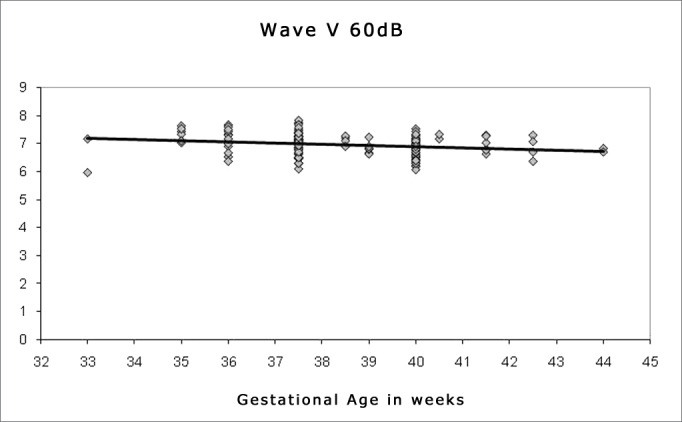
Correlation chart of the gestational age and absolute wave V latency at 60 dBHL. Spearman's correlation coefficient (r=-0.267; p<0.05).

**Figure 5:**
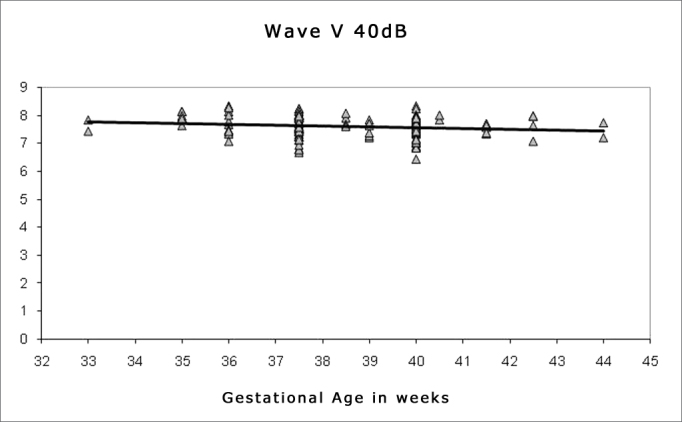
Correlation chart of the gestational age and absolute wave V latency at 40 dBHL. Spearman's correlation coefficient (r=-0.159; p<0.05).

**Figure 6:**
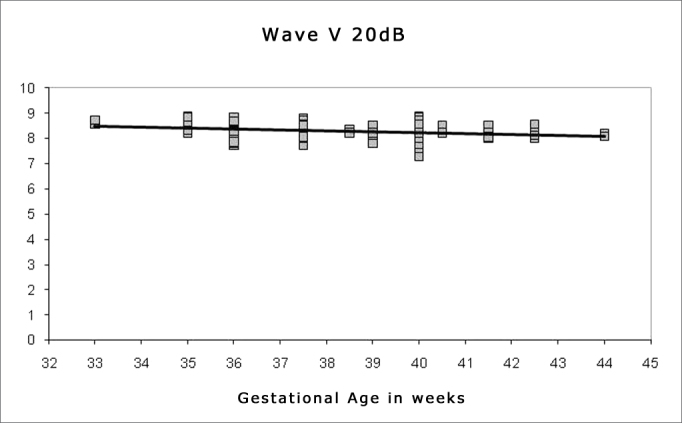
Correlation chart of the gestational age and absolute wave V latency at 20 dBHL. Spearman's correlation coefficient (r=-0.236; p<0.05).

Spearman's correlation coefficient was applied to check the correlation between gestational age and wave I, III and V absolute and interpeak latencies at 80 dBHL, and between gestational age and absolute wave V latency at 60, 40 and 20 dBHL; the results are presented on Figures 1, 2, 3, 4, 5 and 6. There an inverse correlation between gestational age and absolute wave I, III and V latencies at 80 dBHL and wave V at 60, 40 and 20 dBHL (Figs. 6, 7, 8, 9 and 10) in all infants. There was no correlation between gestational age and interpeak latencies.

The comparison of absolute and interpeak latency vales and the reference values for adults revealed a general increase in all components except for the absolute wave I latency, where values were similar to those of adults.^13^

## DISCUSSION

In term and preterm infants the wave V was found at all intensities that were tested up to 20 dBHL. This finding concurs with other published results.^8^, ^19^ Our inclusion criteria discarded outer and middle ear conditions and cochlear diseases. These inclusion criteria justify the presence of wave V even at 20 dBHL, thereby confirming the absence of hearing loss in the study sample.

There were no statistically significant differences in a comparison of absolute wave I, III and V and interpeaks III-V and I-V latencies in the right and left ears. A statistically significant difference was found only in the interpeak I-III, where left ear values were higher than right ear values (Table 2). Other studies have not reported any significant absolute and interpeak latency differences between ears.^7^, ^8^, ^9^, ^10^, ^25^ These authors found that in subjects with normal peripheral hearing, the responses of both ears in brainstem audiometry are similar, as the anatomical structures are part of the brainstem itself, which are used by both ears when a sound stimulus occurs. Munhoz^26^ stated that waves I and II arise ipsilaterally to the stimulus and reflect the action potential of the auditory nerve, whereas waves III, IV and V receive contralateral inputs probably in a greater number than ipsilateral inputs. Both ears will use post-synaptic activities originating in several regions of brainstem auditory pathways when responding to a sound. Thus, subjects with no peripheral disorders, the results of an ear may correlate with the expected results of the opposite ear. One study, however, differed in that lower interpeak intervals and higher amplitudes were found in the right ear of tested subjects, as we found in our sample for the interpeak I-III.^21^

A comparison of wave I, III and V absolute and interpeak latencies in our study sample revealed no statistically significant gender differences at all tested intensities (Table 3). These findings concur with those of other studies of neonates, lactating infants and older children,^7^, ^8^, ^18^ but disagree with other studies of neonates, lactating infants, children and adults where longer latencies were found in males, mostly for absolute wave III and V latencies.^10^, ^17^, ^21^, ^26^, ^27^ The authors explain these findings by the fact that females have faster cochlear responses, which may underlie earlier brainstem responses.^28^, ^29^

Most subjects presented an inter-ear absolute wave V latency difference below 0.4 ms. These findings concur with other published results, which have reported that the inter-ear wave V difference in subjects with symmetrical hearing remains below 0.4 ms.^13^, ^18^, ^19^ Results were similar in children and adults, suggesting that symmetrical responses do not vary with age.^18^

The mean values and standard deviation of waves I, III and V absolute and interpeak latencies concurred with other published studies of term and premature newborn and similar reference values.^2^, ^9^, ^18^, ^19^, ^20^, ^30^, ^31^However, values in premature neonates differed from those in a single study where more prolonged absolute values were found at 80 dBHL in preterm newborn that were evaluated at age 4 months.^7^

The latency differences in some of the BAEP components in the abovementioned studies may be due to device differences. Other authors have stated that devices should be considered in data analysis to achieve reliable results and increase diagnostic accuracy.^32^, ^33^ This possibility is confirmed because the absolute and interpeak wave latency values of term newborn in our sample were close to those found in a control group of another study of similarly aged subjects using the same equipment and parameters as those of the present study.^1^ Furthermore, the maximum and minimum BAEP component results in the present study varied significantly, showing a wide range of normal values (Table 4). A few authors have also described this finding in term and premature newborn and in older children.^8^, ^9^, ^30^, ^34^ Such variability may be due to individual auditory pathway maturation differences, as well as difficulties in defining the conception date (gestational age plus post-natal age) versus the gestational age of the newborn with a greater than two weeks margin of error;^35^, ^36^ during this interval, maturation levels of the auditory transmission time in neonates change rapidly, especially in premature infants.^16^

BAEP may be used to assess the integrity of auditory pathways from the auditory nerve to the brainstem.^10^ The presence of normal wave I, III and V absolute and interpeak latency values at 80 dBHL could be detected in the study sample, as these results were similar to those of other studies of lactating infants in the same age group with no auditory disorders. Normal interpeak intervals and the presence of waves I, III and V at 80 dBHL with normal absolute latency values suggest that auditory pathways are normal up to the brainstem in our study sample. Our inclusion criteria also justify this findings; not only were peripheral hearing disorders discarded, but also the incidence of central auditory disorders in lactating infants with no risk factors for hearing loss is low.

Other researchers of BAEP in premature newborn have also found increased absolute latency and interpeak interval values compared to term newborn, and have suggested that these variables are affected by the maturation process of the hearing system.^7^, ^18^, ^19^ These findings, however, differ from those of a study in which premature neonates with gestational ages ranging from 33 to 36 weeks showed no significant differences in absolute and interpeak latencies compared to term neonates.^37^

An increased absolute latency in premature compared to term newborn may be related to electrical conduction delays because of myelinization of developing auditory pathway structures up to the brainstem; this suggests that the degree of nerve fiber myelinization and immature auditory pathways affects wave latency.^7^, ^18^ This possibility is confirmed by the global delay in absolute and interpeak latencies in the study sample compared to the adult population, as well as the inverse correlation between gestational age and absolute latencies. The latency increase was even more marked in premature newborn, as the maturity level in this group is at an earlier stage compared to term neonates, since this process depends on the gestational age. This has also been reported in other papers.^7^, ^15^, ^19^, ^20^

In relation to interpeak intervals, delays in central conduction times in adults may also be related to changes in myelinization-associated neural conduction velocities and/or changes in synaptic efficiency across the auditory pathway nuclei.^7^, ^9^ The brainstem portion that contains auditory pathways triples in length between the 21^st^ fetal week and the first year of life; the auditory pathway continues to grow until about the third year of life as the brainstem diameter increases.^15^ However, interpeak intervals decrease as pathways grow longer and specialize after birth, which increases conduction velocity at a rate that precisely compensates their physical growth.^15^, ^38^

The inverse correlation between gestational age and absolute latencies shows that as gestational age increases - and the brainstem in the central nervous system matures - there is a continuous decrease in absolute wave latencies in term and preterm newborn. Such decrease relates to the progressive myelinization of central nervous structures, increased axon diameter, improved neural activity synchronism, effective structural connections, and improved synaptic function; all of these factors derive from the maturation process of the central auditory system. These processes yield an improved morphology and reduction in the latency of auditory evoked potential components.^7^, ^20^, ^34^, ^38^ Other studies have also shown a systematic decrease in latencies as a function of increased age.^7^, ^12^, ^17^, ^18^, ^20^, ^30^ Therefore, it may be concluded that gestational age is a factor to be taken into account when interpreting BAEP testing in neonates and lactating infants.

A few studies have shown that the development and maturation of the peripheral auditory system - comprising the outer and middle ear, cochlea and eighth nerve (wave I generating site) - is complete by about 24 weeks gestational age, and that this system is fully formed by the time of birth.^39^, ^40^ The wave I latency increase in premature newborn compared to terms newborn and adults, and the inverse correlation between absolute wave I latency and gestational age are evidence of a cochlear development process and continuous myelinization of the auditory nerve that continue after birth in premature neonates. Maturation of the peripheral auditory system was complete - or became complete within a few weeks - in term infants, given that absolute wave I latency values were similar to normal adult values.

Although absolute wave I latency was decreased depending on the gestational age in term and preterm neonates, the mean absolute wave I latency values at 80 dBHL in term newborn were similar to reference adult values as reported in the literature.^10^, ^13^, ^17^, ^32^ In premature lactating infants, absolute wave I latency values were mildly increased, but were still close to those in subjects aged over 24 months. On the other hand, wave III and V values were significantly higher in lactating infants of our sample, compared with adult reference values.

Given these results, the results show that wave I maturation occurs faster and is complete by birth or within the first few weeks of birth in term births. The wave III - and especially the wave V - generating structures are more central and are under the influence of the maturation period for a longer time frame, reaching adult values at about the second year of life.^7^, ^12^, ^14^, ^15^, ^39^, ^40^ It may be concluded that auditory pathway maturation up to the brainstem progresses in the caudal-rostral direction, as described by Eggermont^41^ and Zimmerman et al.^30^ where the peripheral pathway matures earlier and the rostral portion matures later.

Based on the data, and given the importance of BAEP in the diagnosis of hearing losses in children, our recommendation is that each healthcare unit should establish reference values for its pediatric population, taking into account variables such as sex, gestational age, and the equipment. Such data may be used as a reference value for analyzing the results of very young children to differentiate between expected and altered results at each age group.

## CONCLUSION

The results show that the maturity of the hearing system affects BAEP responses in the newborn. Thus, the gestational age should be taken into account to improve the accuracy of this test in neonates and lactating infants, thereby avoiding erroneous interpretation of the results.
